# The complete chloroplast genome of *Leptodermis scabrida* (Rubiaceae): an endemic shrub in Himalaya-Hengduan Mountains

**DOI:** 10.1080/23802359.2019.1698371

**Published:** 2019-12-12

**Authors:** Ying Zhang, Sheng Chen, Ruijiang Wang

**Affiliations:** a Key Laboratory of Plant Resources Conservation and Sustainable Utilization, South China Botanical Garden, Chinese Academy of Sciences, Guangzhou, China;; b College of Life Sciences, University of Chinese Academy of Sciences, Beijing, China;; c College of Life Sciences, South China Agricultural University, Guangzhou, China

**Keywords:** *Leptodermis*, chloroplast genome, phylogenetic, Rubiaceae

## Abstract

*Leptodermis scabrida* is an important endemic species in Himalaya-Hengduan Mountains. Here, we report the complete chloroplast genome of *L. scabrida*. The cp genome was determined to be 154783 bp in length and the GC content was 37.5%. The sequence included a large single copy (LSC) region of 84190 bp, a small single copy (SSC) region of 17183 bp and two separated inverted regions of 26705 bp, respectively. It contained 132 genes, including 84 protein-coding genes, 38 tRNA genes, and 8 rRNA genes. This complete genome of *L. scabrida* will provide valuable information to resolve the complex phylogeny relationship and to elucidate the mechanism of speciation of *Leptodermis*, as well as for the phylogenetic studies of Rubiaceae.

*Leptodermis scabrida* Hook. is a deciduous shrub with a height of up to 1 meter, which belongs to *Leptodermis* Wall., Rubiaceae (Luo 1999b). *Leptodermis scabrida* is a kind of wild native plant with strong resistance that is mainly distributed in the Himalaya-Hengduan Mountains (Li 1985), which is the biodiversity hotspot. Until now, no genome studies of the genus *Leptodermis* has been reported. Therefore, additional more genomic data are urgently needed to resolve the complex phylogeny relationship and to elucidate the mechanism of speciation of *Leptodermis*, which may also provide new insight into other research in Himalaya-Mountains. Here, we report and characterize the complete chloroplast genome (cp genome) sequence of *L. scabrida* (GenBank accession number: MN686284) for the first time.

The fresh, young and healthy leaves of *L. scabrida* were collected from Bomi County, Linzhi City, Tibet, China (29°53′54.7360″N, 95°39′41.1760″E, 2680 m). The voucher specimen (voucher No.: 888) was deposited in the South China Botanical Garden Herbarium (IBSC), Guangzhou, China. The total genomic DNA was extracted from the silica-gel dried leaves following the modified CTAB method (Doyle and Doyle 1987). Then the genomic library (paired-end, PE = 150 bp) was sequenced on an Illumina Hiseq X Ten platform at Beijing Genomics Institute (Shenzhen, China). Totally 2 Gb sequence reads were obtained and used to assemble the cp genome after filtering and trimming the low-quality reads and adaptor sequences. The complete cp genome assembly was executed on NOVOPlasty 2.6.3 (Dierckxsens et al. 2017) with the default k-mer of 39–59, while Geneious version 11.0.3 (Kearse et al. 2012) was used to annotate the genome. *Galium mollugo* (GenBank accession number: NC_036970) was used as the reference plastid genome for assembling and annotation. The tRNA genes were annotated on ARAGORN (Laslett and Canback 2004).

The complete cp genome of *L. scabrida* was 154783 bp with the typical quadripartite structure of angiosperms, including a small single-copy region (SSC) of 17183 bp, a large single-copy region (LSC) of 84190 bp and a pair of inverted repeats (IRs) of 26705 bp. The genome harbored 132 genes, including 38 tRNA genes, 8 rRNA genes, and 84 protein-coding genes. The overall GC content in the cp genome of *L. scabrida* was 37.50%, which the corresponding value of the SSC, LSC, and IR region were 31.40, 35.40, and 42.80%, respectively.

To identify the phylogenetic position of *L. scabrida* in the Rubiaceae, a maximum likelihood (ML) tree was reconstructed by using RAxML (Stamatakis 2006) with 1000 bootstrap replicates under the GTRGAMMA substitution model. The matrix, used for phylogenetic analysis, included 14 whole cp genome sequences of Rubiaceae species (13 of them were downloaded from GenBank) and was aligned in MAFFT (Katoh and Standley 2013), using *Buddleja colvilei* Hook. f. & Thoms (Loganiaceae) (GenBank accession number: NC_042766) as an outgroup (Figure 1). The phylogenetic analysis was divided into three parts, which was consistent with the subfamily classified (Rubioideae, Cinchonoideae, and Ixoroideae). Most nodes in the chloroplast ML tree were strongly supported. *Leptodermis scabrida* was closely related to *Galium mollugo* L. formed a monophyletic clade with strong bootstrap support, belonging to the subfamily Rubioideae.

**Figure 1.**
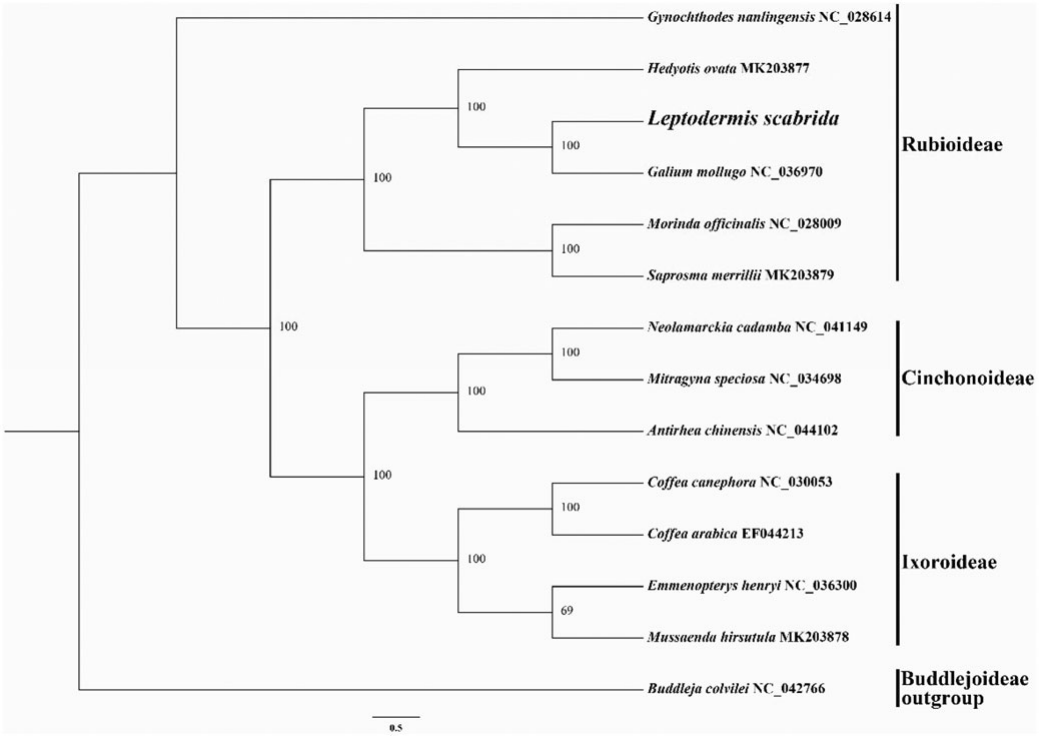
Maximum likelihood tree based on 14 complete chloroplast genomes of Rubiaceae. *Buddleja colvilei* was used as an outgroup. Bootstrap support values were shown at the branches.
